# Minimally Invasive Electro-Magnetic Navigational Bronchoscopy-Integrated Near-Infrared-Guided Sentinel Lymph Node Mapping in the Porcine Lung

**DOI:** 10.1371/journal.pone.0126945

**Published:** 2015-05-20

**Authors:** Hironobu Wada, Kentaro Hirohashi, Takashi Anayama, Takahiro Nakajima, Tatsuya Kato, Harley H. L. Chan, Jimmy Qiu, Michael Daly, Robert Weersink, David A. Jaffray, Jonathan C. Irish, Thomas K. Waddell, Shaf Keshavjee, Ichiro Yoshino, Kazuhiro Yasufuku

**Affiliations:** 1 Division of Thoracic Surgery, Toronto General Hospital, University Health Network, Toronto, Ontario, Canada; 2 Guided Therapeutics, TECHNA Institute, University Health Network, Toronto, Ontario, Canada; 3 Department of Otolaryngology, Head and Neck Surgery/Surgical Oncology, Princess Margaret Hospital, University Health Network, University of Toronto, Toronto, Ontario, Canada; 4 Department of General Thoracic Surgery, Graduate School of Medicine, Chiba University, Chiba, Japan; Peking University People Hospital, CHINA

## Abstract

**Background:**

The use of near-infrared (NIR) fluorescence imaging with indocyanine green (ICG) for sentinel lymph node (SN) mapping has been investigated in lung cancer; however, this has not been fully adapted for minimally invasive surgery (MIS). The aim of our study was to develop a minimally invasive SN mapping integrating pre-operative electro-magnetic navigational bronchoscopy (ENB)-guided transbronchial ICG injection and intraoperative NIR thoracoscopic imaging.

**Methods:**

A NIR thoracoscope was used to visualize ICG fluorescence. ICG solutions in a 96-well plate and *ex vivo* porcine lungs were examined to optimize ICG concentrations and injection volumes. Transbronchial ICG injection (n=4) was assessed in comparison to a traditional transpleural approach (n=3), where after thoracotomy an ICG solution (100μL at 100μg/mL) was injected into the porcine right upper lobe for SN identification. For further translation into clinical use, transbronchial ICG injection prior to thoracotomy followed by NIR thoracoscopic imaging was validated (n=3). ENB was used for accurate targeting in two pigs with a pseudo-tumor.

**Results:**

The ICG fluorescence at 10 μg/mL was the brightest among various concentrations, unchanged by the distance between the thoracoscope and ICG solutions. Injected ICG of no more than 500μL showed a localized fluorescence area. All 7 pigs showed a bright paratracheal lymph node within 15 minutes post-injection, with persistent fluorescence for 60 minutes. The antecedent transbronchial ICG injection succeeded in SN identification in all 3 cases at the first thoracoscopic inspection within 20 minutes post-injection. The ENB system allowed accurate ICG injection surrounding the pseudo-tumors.

**Conclusions:**

ENB-guided ICG injection followed by NIR thoracoscopy was technically feasible for SN mapping in the porcine lung. This promising platform may be translated into human clinical trials and is suited for MIS.

## Introduction

The concept of a sentinel lymph node (SN) is that if SNs do not contain malignant tumor cells, then no metastasis has reached to distant lymph nodes since tumor cells are most likely to spread initially from the primary tumor to a SN [1]. SN mapping currently has been applied to lung cancer surgery using blue dye and radioisotopes [2, 3]. However, blue dye is difficult to distinguish SNs from anthracotic nodes, which has resulted in a 64.4% pooled SN detection rate shown in a recent meta-analysis. Radioisotopes have shown a better detection rate with 84.4%; furthermore, a rate of 90.4% when used in combination with blue dye [2]. It, however, has some critical limitations, including pre-operative invasive procedure, intraoperative shine through effects, lack of real-time images, and further radiation exposure. Additionally, a multicenter phase II trial has shown miserable results, concluding that the difficulty of this technique required an extensive learning curve [3].

Near-infrared (NIR) fluorescence imaging with clinically-approved indocyanine green (ICG) is an emerging modality for image-guided surgery [4] and has been successfully introduced to SN mapping for various organs [5–10]. This has been examined in the porcine lungs [11, 12] and applied to pilot clinical trials for lung cancer patients [13–15]. The NIR imaging appears to be feasible with some advantages over the current traditional methods, including real-time imaging, safety, convenience, and a lower cost [4]. However, it is still under investigation and has not been fully adapted for minimally invasive surgery (MIS). While the previously published NIR fluorescence-guided SN mapping requires intraoperative transpleural ICG injection, this approach is not ideal for MIS, especially when the tumor is indiscernible on thoracoscopy as the lesion can cause conversion to thoracotomy. Therefore, we seek a novel NIR-guided SN mapping technique which would work with MIS. The objective of this study was to develop a minimally invasive SN mapping which integrates pre-operative electro-magnetic navigational bronchoscopy (ENB)-guided transbronchial ICG injection and intraoperative NIR thoracoscopic imaging. This proof-of-concept study has optimized multiple factors determining the fluorescence intensity prior to i*n vivo* porcine studies, demonstrated the feasibility of transbronchial ICG injection for SN mapping, and then implemented ENB-integrated NIR-guided SN mapping in the porcine lung.

## Material and Methods

### NIR fluorescence thoracoscope

A NIR fluorescence thoracoscope (SPY scope, Novadaq Technologies Inc., Ontario) was used to visualize ICG fluorescence. This is a FDA and Health Canada approved rigid thoracoscope with a 0 degree endoscopic view which can provide both color and NIR fluorescence images simultaneously without distortion of the surgical view. This is classified as a class 3R laser with a NIR light of 805±5 nm.

### Quantitative assessment for ICG solutions

ICG (IC-GREEN, Akorn, Illinois) was reconstituted either in distilled water (DW), 5% bovine serum albumin (BSA) (Sigma-Aldrich, Ontario), or porcine plasma (PL), and then different concentrations (5,000, 1,000, 100, 10, 1, 0.1, 0.01, and 0.001 μg/mL) were prepared. The PL was produced by centrifuging fresh porcine blood at 2,000 rpm for 15 minutes [11]. Each ICG solution of 100 μL was placed in a 96-well plate, and observed by the NIR thoacoscope at various distances (1.5, 3, 5, and 7 cm) from the solution surface. Quantitative analysis was performed on the fluorescence videos. Regions-of-interest were drawn on each well using ImageJ [16], and the gray scale values were computed and compared.

### *Ex vivo* porcine lung preparation

*Ex vivo* porcine lungs were employed to evaluate the dimensions of injected ICG in the lung. ICG solution at 100 μg/mL concentration paired with 5%BSA was injected, transpleurally, into the inflated lower lobes. Different volumes of ICG (100, 500, and 1,000 μL) were examined. The injection procedure was the same as the *in vivo* transpleural ICG injection described later. The lung was sliced at the injection point, and observed using the thoracoscope. The fluorescence areas on cross sections were calculated within the merged images by ImageJ and compared.

### *In vivo* NIR fluorescence-guided SN mapping technique

This study was approved by the Animal Care Committee (protocol AUP 2044) at the University Health Network (UHN). All animals were provided humane care in accordance with the policies formulated by the UHN Animal Care Committee, the Animal for Research Act of the Province of Ontario, and the Canadian Council on Animal Care. They were kept in a dedicated facility in our laboratory and provided appropriate food and water by dedicated staff. All animals were euthanized with sodium pentobarbital at the end of experimental procedures.

Male adult Yorkshire pigs (Caughell Farms, Fingal, Ontario) were used. A cocktail of ketamine (20 mg/kg), atropine (0.04 mg/kg) and midazolam (0.3 mg/kg) was administered by intra-muscular injection, and then inhaled isoflurane (5%) was used for the induction of general anesthesia. An endotracheal tube was inserted through a tracheotomy for mechanical ventilation and vital signs were monitored throughout the operation under general anesthesia which was maintained with 2 to 5% isoflurane and 4 L/min of oxygen. After bilateral thoracotomy via a clam shell incision, an ICG solution was injected either transpleurally or transbronchially into the lung and bright lymph nodes were identified using the NIR thoracoscope. The transpleural injection was performed at a 5 to 10mm depth from the lung surface using a 27G tuberculin needle and a surgical clip to minimize the leakage of ICG from the injection point [13]. Transbronchial ICG injection was performed through an aspiration needle catheter (MAJ-65, OLYMPUS MEDICAL SYSTEMS CORP., Tokyo) which contained the ICG solution. The ICG solution was advanced to the tip of the catheter before the injection. A thin or an ultra-thin bronchoscope was used for the injection with fluoroscopy assistance. Endoscopic images confirmed that the needle was in the lung and not in the bronchial lumen. A continuous positive airway pressure of 30cm H_2_O with manual ventilation was employed for a minimum of 3 minutes after ICG injection to facilitate ICG migration into the lymphatic system [14].

Bright lymph nodes were inspected at regular time points for 60 minutes after the ICG injection, preserving the lymphatic system as much as possible; therefore, mediastinal, not hilar, lymph nodes were targeted for this study. Finally, the lymph nodes were exposed by dissecting the pleura and then examined. In addition, all lymph nodes were then resected and examined *ex vivo*. A NIR-positive node was defined as an obviously bright lymph node *in vivo* as well as *ex vivo* when observed from a 3cm or more distance. If the fluorescence could not be detected from a distant view (more than 3cm), the lymph node was defined as a NIR-negative node, even if it had a slight fluorescence detected from a close-up view.

### Transbronchial approach compared to transpleural approach

The transbronchial ICG injection (n = 4) was compared to the transpleural approach (n = 3). The anterior segment of the right upper lobe was selected for the ICG injection (100μL at 100μg/mL) as the lymphatic flow in the lobe predictably lead to a paratracheal node. The volume and concentration of ICG solution was based on the antecedent desk-top and *in vivo* experiment. Autologous PL was used for a coupling agent, in order to reduce background signals in the tracheobronchus [11]. Open thoracotomy was selected instead of thoracoscopic surgery to make the comparison precise and simple. First, it allows intraoperative transpleural ICG injection into completely inflated lungs which mimics the transbronchial approach. In addition, open thoracotomy enables us to confirm an ICG injection point of the lung in both approaches, resulting in an accurate comparison between similar injection points.

Since the NIR fluorescence thoracoscope does not allow real-time quantification of fluorescence signals, semi-quantitative analysis was retrospectively performed to demonstrate the time course of fluorescence intensity of NIR-positive nodes. Fluorescence intensity of targets was assessed on the NIR thoracoscope videos. Multiple frames showing NIR-positive nodes were exported to ImageJ and the gray scale values of regions-of-interest were calculated. As the distance and angle-of-view of the scope directly affects the fluorescence intensity, these factors were kept as consistent as possible when exported for the fluorescence intensity calculation. Since the fluorescence intensity from the background was extremely low and often showed zero, especially when a SN is significantly bright, signal-to-background ratio was not calculated.

### Minimally invasive electro-magnetic navigational bronchoscopy-integrated SN mapping technique

A minimally invasive SN mapping technique integrating pre-operative transbronchial ICG injection and intraoperative NIR fluorescence imaging was validated using 3 pigs as a proof-of-concept study. ICG (100μL at 100μg/mL, paired with PL) was injected into the lower lobes (2 in the right, 1 in the left) transbronchially as described above. Immediately after the injection, unilateral thoracotomy of the injected side was performed followed by NIR-guided SN identification. For the first pig, ICG was injected into the lung parenchyma without the use of the navigation system. In the remaining two cases, an in-house prototype ENB system was used for ICG injection nearby pseudo-tumors. This system has been developed by our team under collaboration with OLYMPUS MEDICAL SYSTEMS CORP. and proven to be feasible for accurate targeting of pseudo-tumors for intraoperative NIR-guided lung nodule localization [17]. A pseudo-tumor was made by a percutaneous injection of 5% agar with a contrast agent under fluoroscopy assistance, and then chest computed tomography (CT) imaging was obtained using a prototype mobile C-arm cone-beam CT (CBCT, PowerMobil, Siemens Healthcare, Erlangen, Germany) which is capable of obtaining intraoperative 3D datasets with sub-millimeter spatial resolution and soft tissue visibility at a low radiation dose [18]. The field of view encompasses 20x20x15 cm^3^ which is sufficient to capture the volume of interest of the lung and create a navigational path for targeting. Thereafter, the flexible bronchoscope with an electro-magnetic sensor (Aurora, Northern Digital Inc., Waterloo, Ontario) on its tip was inserted into the airway through an endotracheal tube for ICG injection. Bronchoscopic images were co-registered in real time to 3D CBCT datasets using in-house software developed for image-guided surgery [19]. The software provided a navigational path on virtual images which guided the operator to the target, so that accurate ICG injection was achieved. The accuracy of the injection site was confirmed by fluorescence images on cross sections of the pseudo-tumor in the lung.

### Statistical analysis

Graphs were created and statistics were calculated by GraphPad Prism software (version 5.01, La Jolla, CA). The Kruskal-Wallis analysis of variance test followed by Dunn’s multiple comparison test was used for the comparisons of more than two variables. All p-values were based on a two-sided hypothesis, a p-value of <0.05 was considered to have statistical significance.

## Results

### Optimization of multiple factors determining the fluorescence intensity

**Fig 1:**
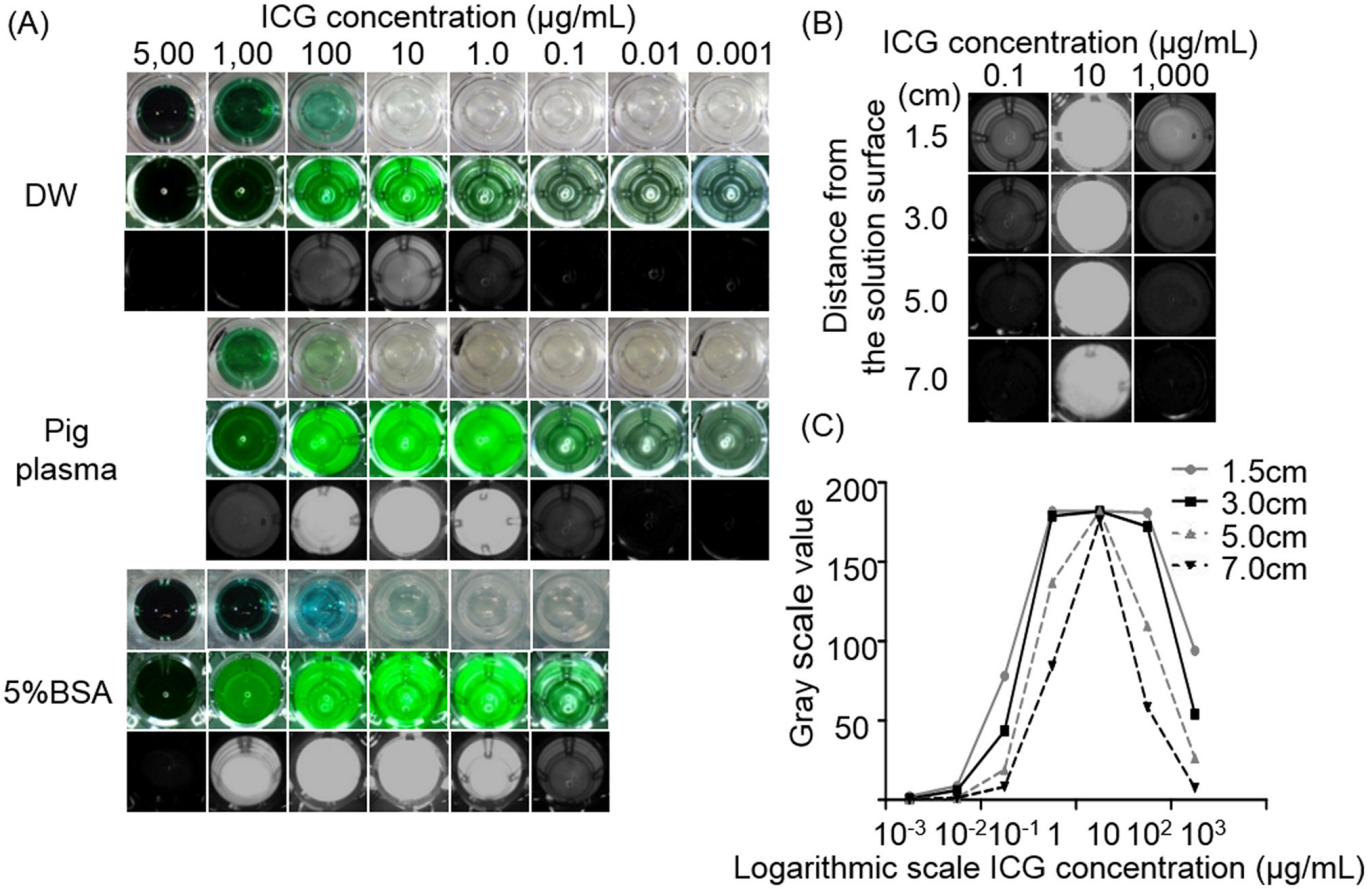
Multiple factors determine the ICG fluorescence intensity. (A) Autologous porcine plasma (PL) and 5% bovine serum albumin (BSA) enhanced the ICG fluorescence intensity, with the brightest intensity at 10 μg/ml (at 3cm). Top: normal images, middle: merged images, bottom: fluorescence images. (B, C) Fluorescence intensity depended on the distance from the objects. ICG was paired with PL.

As previous studies have demonstrated [11], both PL and BSA dramatically enhanced ICG fluorescence intensity. Regardless of the type of coupling agent, 10 μg/mL concentrated ICG showed the brightest fluorescence among the various concentrations. (Fig 1A). The distance between the NIR scope and ICG surface is another principle factor to determine the ICG fluorescence intensity. Even though the same ICG solution was observed, the fluorescence intensity got weaker when the thoracoscope was far away from the ICG solutions. However, 10 μg/mL concentrated ICG showed intense fluorescence even at a distance of 7 cm (Fig 1B and 1C).

**Fig 2:**
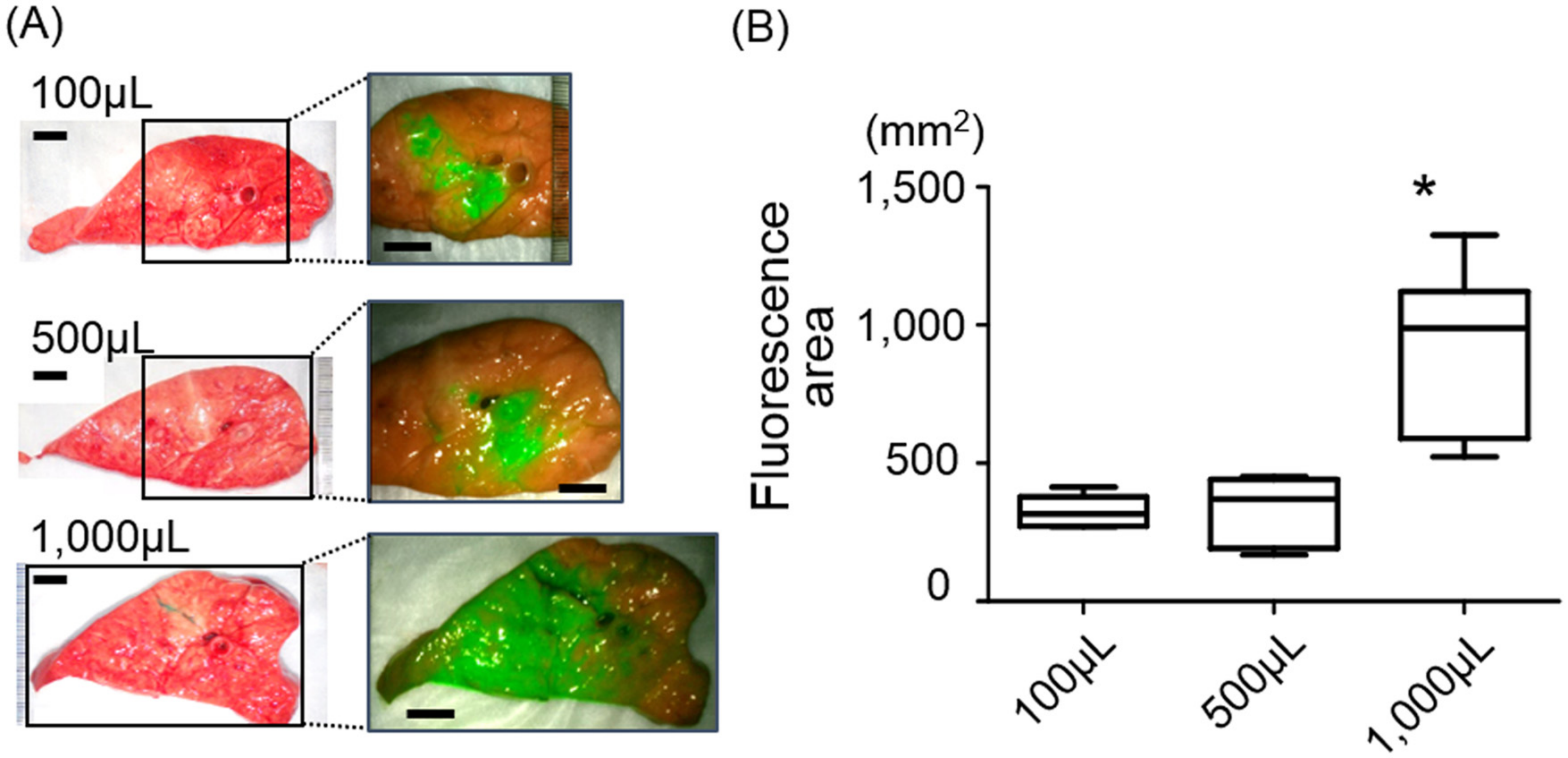
Injection volume was optimized using *ex vivo* porcine lungs. (A) The lung cross sections showed the dimensions of injected ICG (100 μg/ml, paired with 5%BSA) which depended on the volume of the injection. Scale bars show 10 mm. (B) ICG injection of 1,000 μL resulted in significantly larger ICG florescence area compared to the other 2 doses (*p = 0.0033, Kruskal-Wallis). Each box consists of both sides of cross sections of 3 lungs (n = 6).

Regarding the injection dose, the fluorescence surface area revealed no significant difference between the 100 and 500 μL injection, but the dose of 1,000 μL showed a significantly larger area of ICG on the lung cross sections (n = 6, p = 0.0033) (Fig 2). This would suggest that a 1,000 μL dose is an excess required dose for pin-point marking, likely leading to lower accuracy of SN mapping due to an incorrect trace of lymphatic flow which originated from other areas without a tumor.

### *In vivo* comparison between the transbronchial and transpleural approaches

**Fig 3:**
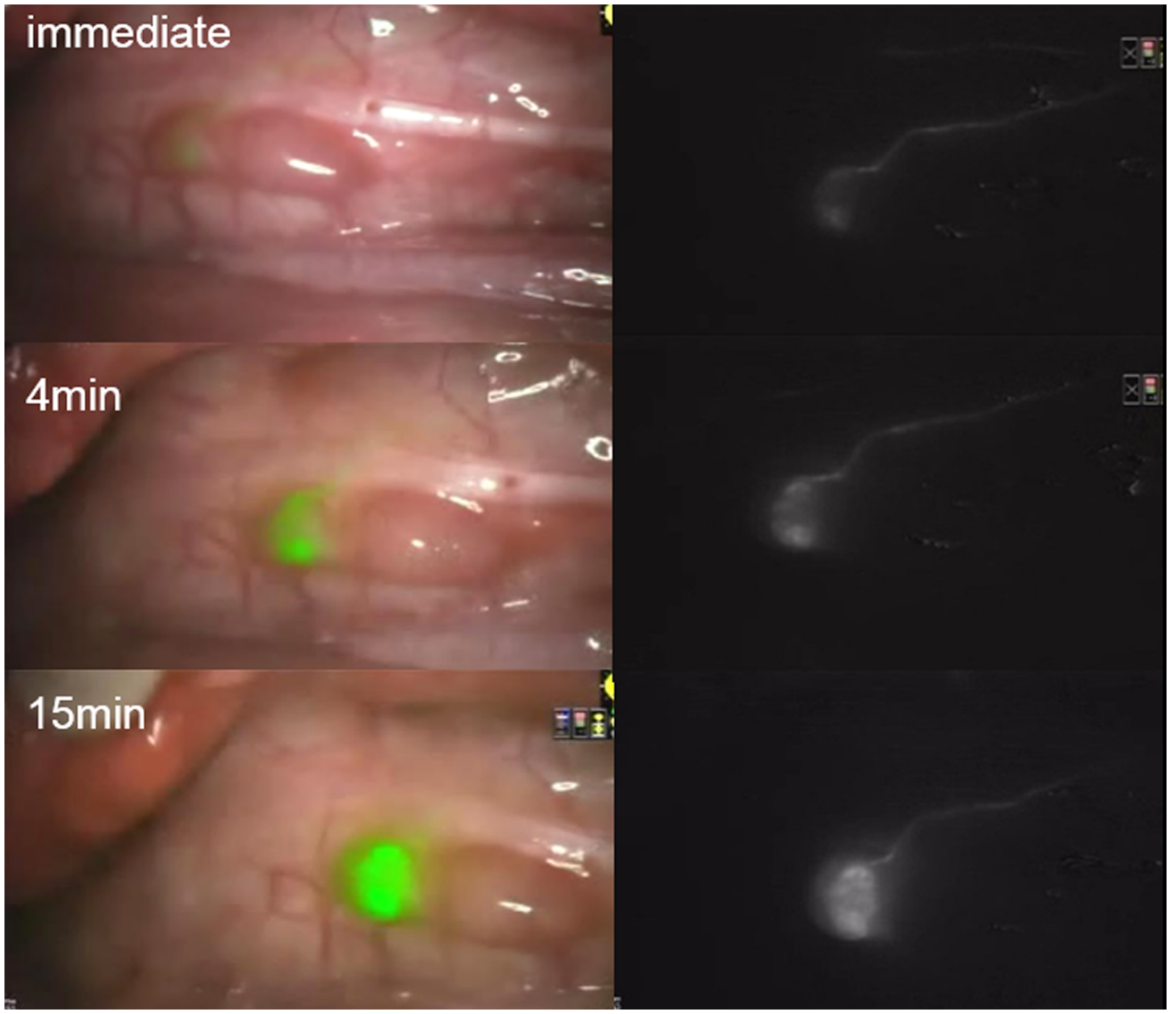
Successful sentinel lymph node detection using the near-infrared fluorescence imaging. One of the subcarinal lymph nodes was successfully identified as a sentinel lymph node immediately after transpleural ICG injection (100 μg/ml, 100 μL) into the right lower lobe. The afferent lymph vessel was clearly visualized as well, showing a high contrast when compared to the background. Left: merged images, Right: fluorescence images.

After technical optimization of the NIR image-guided SN mapping (Fig 3), transbronchial ICG injection was compared with the traditional transpleural approach. Table 1 shows the time course trend of the number and fluorescence intensity of NIR-positive lymph nodes after ICG injection. The detection rates of SNs were both 100%. In 5 cases, the first NIR-positive lymph node was identified within 5 minutes. In the two transbronchial approach cases, the ICG fluorescence was initially identified at the 15 minute inspection, yet demonstrated by retrospective review of NIR image records at 5 minutes. Fluorescence at NIR-positive nodes was persistent until 60 minutes after injection in all cases; however, some transbronchial cases showed slight attenuation of the fluorescence intensity over time. Additionally, the transbronchial ICG injection tended to have less fluorescence in both lymph nodes and tracheobronchus than the transpleural approach. This would suggest that the transbronchial injection requires a larger dose than the transpleural one for SN mapping, though it shows a high success rate. Although the injected ICG flowed into the tracheobronchus in 4 cases (two cases in each approach) and semi-quantitative fluorescence intensity of the tracheobronchus was higher than bright lymph nodes at 60 minutes, SN identification was possible by completely exposing lymph nodes as well as examining *ex vivo* fluorescence. The bright lymph nodes *in vivo* also showed bright fluorescence *ex vivo*.

**Table 1:** The time course trend of the number of NIR-positive lymph nodes after ICG injection into the right upper lobe in the porcine lung.

Cases^a^	The number of NIR-positive lymph nodes						Semi-quantitative fluorescence intensity		
	5min	15min	30min	60min	Post-pleural dissection	*Ex vivo* ^b^	Bright LNs at the initial detection^c^	Bright LNs at 60min^c^	Tracheobronchus at 60min^d^
**Transpleural 1**	1	2	2	3	3	3/ 9	25.7 (8.5–42.0)	47.7 (14.8–94.4)	-
**Transpleural 2**	1	2	2	2	3^e^	3/ 9	43.8 (16.9–70.8)	59.9 (9.9–109.9)	101.1
**Transpleural 3**	1	1	1	1	1	1/ 10	35.7	82.0	68.0
**Transbronchial 1**	1	1	1	1	1	1/ 7	39.4	18.2	45.0
**Transbronchial 2**	1^f^	1	1	1	2^e^	2/ 9	29.3^g^	17.7	-
**Transbronchial 3**	3	4	4	4	4	4/ 7	93.2 (32.7–129.5)	79.0 (56.3–124.9)	-
**Transbronchial 4**	1^f^	1	1	1	1	1/ 9	14.0^g^	18.6	41.7

The number of NIR-positive lymph nodes and semi-quantitative fluorescence intensity were described.

^a^Seven pigs were used and each pig underwent one ICG injection into the right upper lobe.

^b^The number of bright lymph nodes identified in the *ex vivo* examination (numerator) and the total number of dissected lymph nodes (denominator) are shown.

^c^Represents mean (range) of the fluorescence intensity among the bright NIR-positive lymph nodes.

^d^Tracheobronchus fluorescence was initially identified within 5 minutes in all 4 cases. The other 3 cases showed no fluorescence at the tracheobronchus.

^e^One bright lymph node located deep in the mediastinal fat was identified during lymph node dissection.

^f^The extremely slight ICG fluorescence was identified by retrospective review of NIR image records at 5 minutes, not *in vivo* examination.

^g^Fluorescence intensity at 15 minutes.

### Multi-modal minimally invasive SN mapping technique

**Fig 4:**
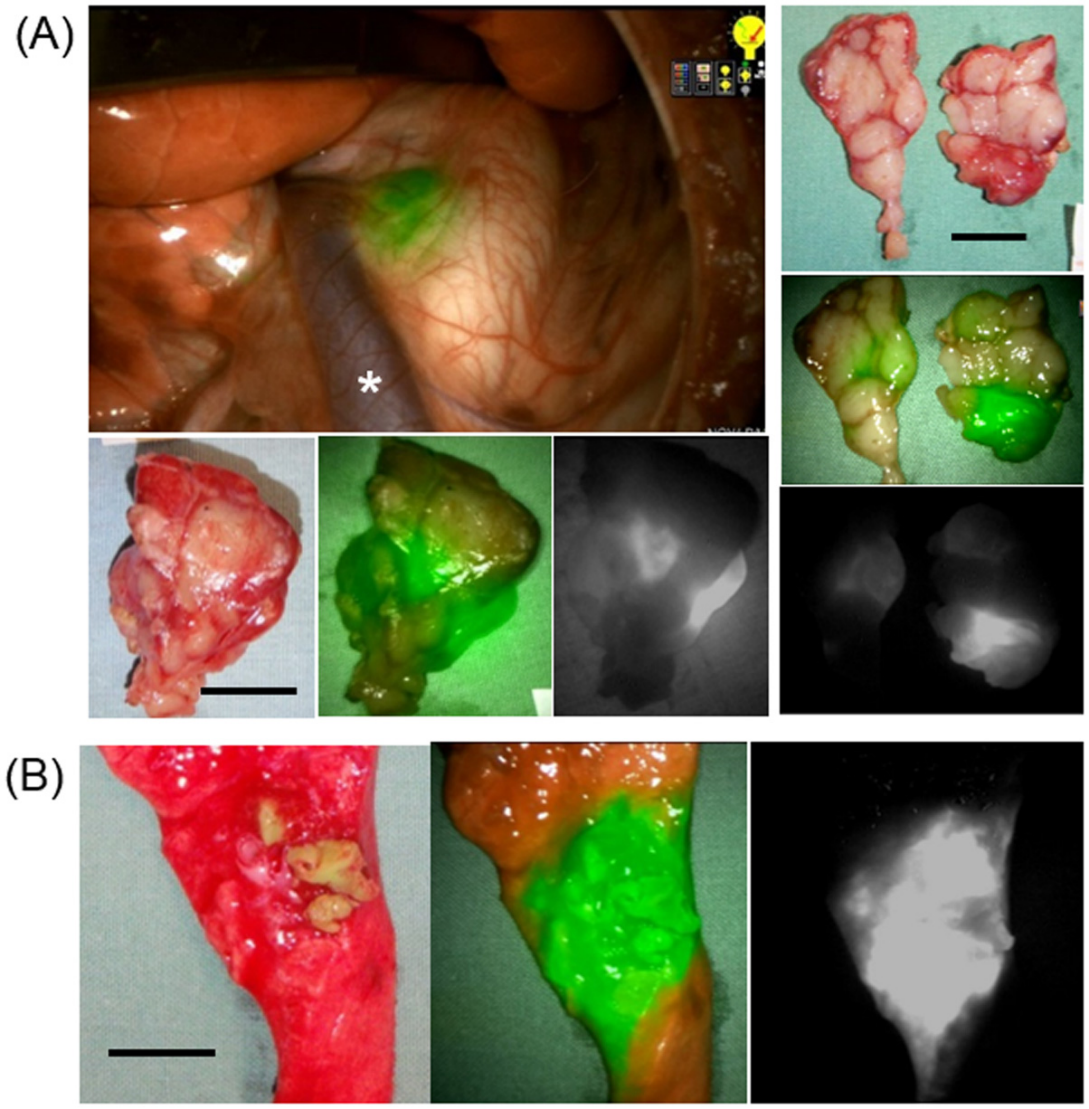
Successful sentinel lymph node identification after pre-operative tansbronchial ICG injection. Electro-magnetic navigational bronchoscopy-guided transbronchial ICG injection (100 μg/ml, 100 μL) into the left lower lobe enabled the SN node identification by the NIR thoracoscope. (A) The left paratracheal lymph node was recognized as the SN. Left top: intraoperative NIR image, left bottom: *ex vivo* examination of the SN, right: *ex vivo* examination of the lymph node cross sections which showed select bright portions. Asterisk shows hemi-azygos vein. (B) The navigation guidance led the bronchoscope to the target accurately. The scale bar shows 10 mm.

Pre-operative transbronchial ICG injection identified NIR-positive lymph nodes in all 3 cases at the first thoracoscopic inspection within 20 minutes post-injection. Injection in the right lower lobe led to SN identification in paratracheal lymph nodes on right side in the first case and both sides in the second case. The left lower lobe injection identified a left paratracheal lymph node as a SN. The pseudo-tumors were created peripherally within 2 cm from the lung surface with an approximate size of 1 cm in diameter. In the two cases tested, the ENB allowed accurate targeting of the pseudo-tumors, demonstrated on the lung cross sections showing the fluorescence surrounding the tumor (Fig 4). The ENB-guided ICG injection was achieved within 15 minutes, including the CBCT image acquisition for 1 minute, a bronchial pathway processing for 5 minutes, and bronchoscopy procedure for approximately 7 minutes.

## Discussion

We have demonstrated that the ENB-integrated NIR-guided SN mapping is feasible in the porcine lung. One of the advantages of this platform is that it works well with MIS for lung cancer patients, including robotic surgery. ICG setting was optimized based on the initial experiments and the NIR thoracoscope clearly visualized ICG fluorescence in lymph nodes as well as lymph vessels in the porcine models. The transbronchial ICG injection led to a similar outcome as the traditional transpleural approach. Furthermore, the ENB enabled precise ICG injection into peri-tumoral lung parenchyma in the porcine lung within an acceptable additional time. This multi-modal minimally invasive SN mapping technique showed promising results and may be clinically translated into minimally invasive lung cancer surgery.

Since complete lymph node dissection adds little morbidity to lung cancer surgery [20], the benefits yielded by SN mapping for lung cancer patients differs from breast cancer and malignant melanomas. Intraoperative SN mapping can offer evidence for the selection of segmentectomy candidates. With the use of low-dose CT screening, early-stage lung cancer will be more often detected. Sublobar resection is a treatment option and has shown similar results to lobectomy for patients with early-stage (<2cm) disease [21]. Moreover, two ongoing randomized phase III clinical trials in North America [22] and Japan [23] may provide additional evidence for performing sublobar resection in early-stage lung cancer. Sublobar resection has a great potential to become a standard option of care in select patients and precise lymph node diagnosis, including N1 nodes surrounding the responsible segmental bronchus and ‘skip metastasis’ of N2 nodes (reported 20.2% to 38% in pN2 disease [24]), is a key for its success. Intraoperative SN mapping followed by pathological analysis on frozen sections of SNs has been demonstrated to facilitate decision making for successful segmentectomy [25]. Additionally, the detection of micrometastasis in SNs may contribute to proper staging and a survival benefit [26, 27]. With a recent trend toward MIS and expanding demand for segmentectomy, we expect that the proposed SN mapping technique will be increasingly required for selecting appropriate candidates for sublobar resection.

The concentration and injected dose of ICG solution are of importance for precise SN identification. A high concentration leads to quenching, and a low concentration induces extremely weak fluorescence. The desk-top study showed the most intense fluorescence at 10 μg/mL and the lowest detectable concentration at 0.1 μg/mL. We used this to determine the proper concentrations for the following *in vivo* study, which was 100 μg/mL, as the injected ICG solution would be diluted on the way to SNs. The total dose of injected ICG in the previous lung cancer clinical trials ranges from 3.8 μg [14, 15] to 10,000 μg [13]. The latest phase I dose-escalation trial has shown that a greater than 1,000 μg yielded 89% of SN detection rate, while a less than 600 μg injection resulted in a lower detection rate of only 20% [15]. High dose of ICG appears to contribute to the successful SN identification in human lungs; however, a large volume of injected ICG spreads throughout a huge portion of the lung, which may cause false tracking of the lymphatic system from the lung without a tumor. Moreover, it may induce a brighter background due to extra ICG which flows into the bronchus. Dose optimization in the human lung is mandatory for using our technique in future clinical trials.

The best coupling agent for ICG during SN mapping remains controversial. Premixing with albumin or plasma enables increased fluorescence intensity; however, when ICG alone is injected into the human lung, it binds to various proteins, resulting in a bright fluorescence. A farther distance from the injection site to the SNs also can cause even more ICG binding to proteins within the lymphatic fluid, leading to successful SNs detection which is equivalent to that of ICG coupled with albumin in a porcine lung study [12]. Indeed, some groups have shown successful SN detection using ICG alone in lung cancer patients [13, 28]. We eventually selected autologous PL to reduce background signal from the bronchus [11]; however, high background fluorescence was detected in 4 out of 7 cases. This is likely because the porcine right upper lobe is relatively small and there may be large bronchioles connecting to the tracheobronchus at the injection depth. Alternatively, another reason could be that the transbronchial injection cannot avoid a slight leakage from the injection point, even though the needle was inserted into the lung.

Transbronchial tracer injections have been attempted during SN mapping for lung cancer patients using a radioisotope [29] or a CT contrast agent [30], showing the SN detection rate of 95.0% and 92.3%, respectively. The ‘pre-operative’ transbronchial injection is less invasive, and is more effective, physiologically, in facilitating tracer migration into the lymphatic system within the lung rather than the intraoperative transpleural approach, in which the tracer is injected into the partially deflated lung. Compared to a CT contrast agent which allows for pre-operative lymphangiogram, ICG has an advantage of real-time intraoperative imaging along with a thoracoscopic surgical view. Furthermore, the navigation-guided transbronchial approach benefits successful ICG injection into the peri-tumoral lung, especially when a target tumor is small and located in the deep parenchyma while the transpleural approach has difficulty with ICG injection for such cases. Both detection rates were 100% (Table 1); however, the transbronchial approach appears to take more minutes to identify SNs and has less bright lymph nodes. This would be attributed to the difference of the total amount of ICG migration into lymphatic system. A leakage from the injection point on the bronchial wall is inevitable and may cause less ICG migration. A positive airway pressure immediately after the injection is hence required to enhance the ICG migration into lymphatic system. Technically, the transbronchial approach may cause a slight leakage from the tip of the needle on the way to the target as the aspiration needle catheter needed to be filled up with ICG solution and loaded in the accessory channel of the scope in advance. This may have resulted in less than a 100 μL injection, leading to lower fluorescence than the transpleural approach, even though the SN detection rate was 100%.

In a future clinical trial, all of the proposed procedures will be performed in an operating room. Under general anesthesia, a patient will undergo ENB for ICG injection into the peri-tumoral lung parenchyma followed by video-assisted or robotic lung surgery. Mediastinal lymph nodes will be initially assessed by the NIR thoracoscope through a thoracic port which is expected to be achieved within 30 to 45 minutes after the injection which is sufficient for the ICG to reach the SNs. In the course of segmentectomy, hilar and intrapulmonary lymph nodes will also be assessed and bright lymph nodes will be removed. All resected lymph nodes will be assessed by the NIR thoracosocope *ex vivo* and NIR-positive nodes will be pathologically examined. When intraoperative pathological examination on frozen NIR-positive nodes shows metastatic cells, lobectomy will be performed instead of segmentectomy. After lung resection, remaining lymph nodes in the chest will be observed with the NIR thoracoscope. The resected tumor in the lung will be opened and examined to confirm that the ICG was precisely injected into the peri-tumoral lung.

Several limitations with this technique were identified. First, ICG spread diffusely to multiple lymph nodes and lymphatic networks apart from the SNs, even though the fluorescence intensity was not high. Actually, transpleural ICG injection into right lower lobe identified bright fluorescence in a #4L in some cases. This would suggest that the hydrodynamic diameter of ICG is relatively small even after coupling with plasma, causing it to pass through the SNs. As a 3cm distance was set as a cut-off value in this study, future clinical studies may require an even better clinical definition of a SN to determine the accurate SNs. Additionally, the NIR signal from the SNs was visible within a few millimeters from the surface, thereby the sufficient exposure of the lymph nodes by dissecting the pleura is fundamental for precise SNs identification, which would also be effective in avoiding false positive cases due to the fluorescence from the lymphatic networks surrounding the SNs. Lastly, the ICG retention rate remains unclear later than one hour post-injection. Even though a few bright lymph nodes showed a slight attenuation of the fluorescence over time, bright lymph nodes with high fluorescence intensity maintained their fluorescence stability for 60 minutes after injection. Provided that a sufficient dose of ICG migrates into the lymphatic system, the ICG fluorescence would remain in the SNs for over 60 minutes. This should be clarified in future clinical trials.

The lack of a real-tumor model in the porcine lung hampers the identification of tumor-related real SNs. However, the proposed technique is feasible in the clinically relevant porcine lung, which can allow initial translation into human clinical practice. In addition, porcine lungs do not necessarily have N1 lymph nodes, especially in the right upper lobe, which was used for the comparison study. We hence targeted N2 nodes and have not dissected out the segmental bronchus to see the N1 nodes in this study. Since our target includes N1 nodes, we plan to evaluate those nodes in the future clinical trial.

In conclusion, the SN mapping using pre-operative ENB-guided transbronchial ICG injection with intraoperative NIR imaging was feasible in the porcine lung. This platform has been optimized and validated for clinical use, especially for minimally invasive lung surgery. This multi-modal minimally invasive SN mapping technique can potentially be translated into human clinical trials using minimally invasive lung surgery which will be required more frequently in the future.
